# Indirect Flow Diversion for Off-Centered Bifurcation Aneurysms and Distant Small-Vessel Aneurysms, a Retrospective Proof of Concept Study From Five Neurovascular Centers

**DOI:** 10.3389/fneur.2021.801470

**Published:** 2022-01-06

**Authors:** Stefan Schob, Richard Brill, Eberhard Siebert, Massimo Sponza, Marie-Sophie Schüngel, Walter Alexander Wohlgemuth, Nico Götz, Dirk Mucha, Anil Gopinathan, Maximilian Scheer, Julian Prell, Georg Bohner, Vladimir Gavrilovic, Martin Skalej

**Affiliations:** ^1^Abteilung für Neuroradiologie, Klinik & Poliklinik für Radiologie, Universitätsklinikum Halle (Saale), Halle (Saale), Germany; ^2^Institut für Neuroradiologie, Charité - Universitätsmedizin Berlin, Berlin, Germany; ^3^Angiography and Interventional Radiology Unit, Department of Radiology, Azienda Sanitari Universitaria Friuli Centrale, Udine, Italy; ^4^Institut für Radiologie und Neuroradiologie, Heinrich-Braun-Klinikum, Zwickau, Germany; ^5^Department of Diagnostic Imaging, National University Hospital, Singapore, Singapore; ^6^Klinik & Poliklinik für Neurochirurgie, Universitätsklinikum Halle, Halle (Saale), Germany

**Keywords:** bifurcation aneurysms, indirect flow diverting, slipstream effect, distant small-vessel aneurysms, deconstruction over time

## Abstract

**Background:** Treatment of cerebral aneurysms using hemodynamic implants such as endosaccular flow disruptors and endoluminal flow diverters has gained significant momentum during recent years. The intended target zone of those devices is the immediate interface between aneurysm and parent vessel. The therapeutic success is based on the reduction of aneurysmal perfusion and the subsequent formation of a neointima along the surface of the implant. However, a subset of aneurysms–off-centered bifurcation aneurysms involving the origin of efferent branches and aneurysms arising from peripheral segments of small cerebral vessels–oftentimes cannot be treated *via* coiling or implanting a hemodynamic implant at the neck level for technical reasons. In those cases, indirect flow diversion–a flow diverter deployed in the main artery proximal to the parent vessel of the aneurysm–can be a viable treatment strategy, but clinical evidence is lacking in this regard.

**Materials and Methods:** Five neurovascular centers contributed to this retrospective analysis of patients who were treated with indirect flow diversion. Clinical data, aneurysm characteristics, anti-platelet medication, and follow-up results, including procedural and post-procedural complications, were recorded.

**Results:** Seventeen patients (mean age: 60.5 years, range: 35–77 years) with 17 target aneurysms (vertebrobasilar: *n* = 9) were treated with indirect flow diversion. The average distance between the flow-diverting stent and the aneurysm was 1.65 mm (range: 0.4–2.4 mm). In 15/17 patients (88.2%), perfusion of the aneurysm was reduced immediately after implantation. Follow-ups were available for 12 cases. Delayed opacification (OKM A3: 11.8%), reduction in size (OKM B1-3: 29.4%) and occlusion (D1: 47.1%) were observable at the latest investigation. Clinically relevant procedural complications and adverse events in the early phase and in the late subacute phase were not observed in any case.

**Conclusion:** Our preliminary data suggest that indirect flow diversion is a safe, feasible, and effective approach to off-centered bifurcation aneurysms and distant small-vessel aneurysms. However, validation with larger studies, including long-term outcomes and optimized imaging, is warranted.

## Introduction

Technical limitations of conventional endovascular aneurysm treatment, most importantly coiling with and without the help of assistive devices, have triggered the development and clinical use of flow-modulating implants, such as endosaccular flow disruptors and endoluminal flow-diverting stents (1–3). Both classes of devices are based on a dense mesh of braided wires that cover the aneurysm neck, whereby endosaccular flow disruptors act from within the aneurysm, and flow-diverting stents operate from within the parent artery (1). The common therapeutic tenets are (a) inducing thrombosis within the aneurysm and (b) creating complete remodeling of the parent artery *via* providing a solid scaffold for the development of a neointima at the aneurysm-parent artery interface (4–6). Each class of hemodynamic implant has a specific aptitude for certain types of aneurysms–endosaccular flow disruptors have proved to be especially valuable for the treatment of wide-necked bifurcation aneurysms at and distal to the Circle of Willis (3, 7), whereas flow-diverting stents are considered the treatment of choice for wide-necked sidewall aneurysms (8) and non-saccular aneurysms (9–11). Flow-diverting stents have also been used for off-label indications, such as the treatment of bifurcation aneurysms (12–14), but unsatisfactory aneurysm occlusion in a number of cases, together with ischemic and hemorrhagic complications, remains a concern according to some investigators (15, 16). In fact, hemodynamic aspects of bifurcation aneurysms differ substantially and must be evaluated carefully to select the most suitable therapeutic strategy for the individual patient. In case the aneurysm represents spatially distinct, broad-based outpouching of a bifurcating main stem and does not involve the smaller efferent branches of the bifurcation, an endosaccular flow disruptor is a viable and potentially preferable option, as it allows to functionally separate and occlude the aneurysm without affecting the afferent or efferent segments of the bifurcation. If the aneurysm is not centered at the bifurcation but arises slightly distal to it and involves the origin or even a more peripheral portion of a small efferent branch, endosaccular flow disruptors cannot be applied safely, because these dependent vessels are at risk of occlusion. The same applies for aneurysms arising from a peripheral segment of a small cerebral vessel, for example, the anterior communicating artery, M2–M3 branches of the middle cerebral artery, or the cerebellar arteries. In such cases, the concept of implanting a flow-diverting stent proximal to the aneurysm–indirect flow diversion–may represent the most suitable therapeutic option (17, 18). Currently, there is only anecdotal evidence on the application of indirect flow diversion. As a consequence, this study aims to report the experiences of five neurovascular centers, specifically focusing on the feasibility and efficacy of the approach, thromboembolic or hemorrhagic complications, and early aneurysm occlusion rates.

## Materials and Methods

### Ethics Approval

This retrospective study was approved by the ethics committee of the University Hospital Halle/Saale, Germany (IRB00011721 Faculty of Medicine, Martin-Luther-University Halle-Wittenberg).

### Study Design

The study was designed as a multicenter, single arm retrospective analysis. The following neurovascular centers contributed to the study: University Hospital Halle (*n* = 5), Heinrich-Braun-Hospital Zwickau (*n* = 1), University Hospital Udine (*n* = 7), National University Hospital Singapore (*n* = 1), and the Charité Berlin (*n* = 3). Patient data, aneurysm properties, interventional details, and technical as well as clinical complications, together with early angiographic follow-ups, were reviewed. Table 1 summarizes the relevant data.

**Table 1:** Summary of all included cases.

**Patient-No**.	**Age**	**Aneurysm localization**	**Neck width in mm**	**Dome width in mm**	**Dome height in mm**	**Implanted flow diverter**	**Proximal landing zone**	**Distal landing zone**	**Distance from FD to aneurysm in mm**	**OKM immediately after FD**	**OKM last available**
1^a^	57	Left proximal PICA	2.7	4.9	3.8	FRED 4 × 12/18 mm	V4 proximal to the PICA	V4 distal to the PICA	2.0	A1	A1 (no FU yet)
2^b^	72	Right proximal AICA	2	4.5	6.3	Pipeline Flex Shield 4.75 × 14 mm	BA adjacent to V4 confluens	BA–middle third	2.3	A3	A3 (no FU yet)
3^b^	75	Left P1-P2-junction	4.2	6.4	4.9	FRED Jr. 2.5 × 13 mm	P1 segment	P2 segment	1.5	A3	A1 (4 months & 5 months)
4^c^	53	M2: superior trunk	4	5.6	3.4	P48MW_HPC 2 × 9 mm	Distal M1 segment	Proximal M3 segment	1.0	A2	D1 (4 months)
5^d^	58	Left proximal PICA	4.2	4	3	Surpass streamline 3 × 20 mm	V4 proximal to the PICA	V4 distal to the PICA	2.0	A1	D1 (6months & 4years)
6^c^	43	AcomA	3.3	7	7	Silk vista baby 2.5 × 15 mm	A1	Proximal A2	2.4	A3	D1 (4 months)
7^e^	39	MCA-M1	2.2	5.7	5	PED3 vantage with shield technology 2.5 × 12 mm	Distal M1	Distal M1	2.0	B2	B2 (no FU yet)
8^c^	63	Right proximal A1 segment	2.6	7.7	10.6	Silk Vista Baby 2.75 × 15 mm	ICA communicating segment	Right M1 segment	2.1	A3	D1 (3months)
9^c^	55	Right posterior communicating artery	1.3	1.9	1.3mm	Derivo 4.0 × 15 mm	ICA communicating segment	Right M1 segment	1.6	A3	B3 (3months)
10^f^	68	Left SUCA	1.6	2.3	3.9	PED 2 Shield 2.75 × 20 mm	BA: distal third	Left P1 segment	1.1	A2	B3 (4months)
11^g^	63	Right proximal PICA	5.1	3.8	5.2	FRED jr. 3.5 × 22 mm	V4 proximal to the PICA	V4 distal to the PICA	2.1	A2	D1 (18 months)
12^g^	71	Distal M1: origin of lateral fronto-orbital artery	4.8	10.2	8.3	FRED jr.3 × 19 mm	Middle M1	Proximal M2	1.6	A2	D1 (6 months)
13^g^	70	Left M2-M3-segment	5	4.7	3.9	Silk vista baby 2.75 × 15 mm	Inferior trunk: distal third	Parietal artery	0.6	A2	D1 (16 months)
14^g^	66	Right proximal AICA	4.8	3.3	2.4	Silk vista 4 × 15 mm	BA: proximal third	BA: middle third	1.0	A3	D1 (4months)
15^g^	77	Right M1-M2-segment	6.7	10.7	6.7	P48MW_HPC 3 × 15 mm	M1: middle third	M2: inferior trunk (dominant branch)	2.4	A2	B2 (6 months)
16^g^	35	Left proximal PICA	4.3	9.2	4.5	FRED X 4 × 18 mm	V4 proximal to the PICA	Distal V4	0.4	A3	A3 (no FU yet)
17^g^	63	Right proximal A1	3	5.6	6.6	Silk Vista 4 × 20 mm	Right C6 segment	Right M1 segment	1.9	B1	B1 (no FU yet)

*Dual anti-platelet therapy*:

a *9 months ASA 100 mg and Clopidogrel 75 mg daily, followed by ASA only lifelong*.

b *4 months ASA 100 mg and Clopidogrel 75 mg daily, followed by ASA only lifelong*.

c *6 months ASA 100 mg and Ticagrelor 90 mg twice a day, followed by ASA only lifelong*.

d *4 months ASA 100 mg and Ticagrelor 90 mg twice a day, followed by ASA only lifelong*.

e *12 months ASA 100 mg and Prasugrel 30 daily, followed by ASA lifelong*.

f *6 months ASA 100 mg and Clopidogrel 75 mg daily, followed by ASA only lifelong*.

g *3 months ASA 100 mg and Clopidogrel 75 mg, followed by ASA only lifelong*.

*Triaxial endovascular access in eleven cases (patients 1, 2, 4, 5, 6, 7, 8, 9, 10, 13, 14)*.

*Guiding catheter: Neuron Max 088 (Penumbra) or the Benchmark 071 (Penumbra) or the Envoy 6F (Codman)*.

*Distal access catheter: Sofia 5F (Microvention), Sofia EX (Microvention), CAT 5 (Stryker)*.

*Biaxial endovascular access in six cases (patients 3, 11, 12, 15, 16, 17)*.

*Guiding catheter: Envoy 6F or Envoy 7F (Codman)*.

### Endovascular Procedure and Antiplatelet Regimen

All treatments were performed in general anesthesia using biplane digital subtraction angiography (DSA) suite. Arterial access was gained via the right femoral artery. Dual anti-platelet treatment (DAPT) was used in all cases to prevent thromboembolic complications and was performed according to each center's individual regimen. The dosage and duration of the medication for each patient are shown in Table 1. Platelet function testing was not mandatory for our analysis.

### Procedure Assessment, Radiological, and Clinical Follow-Up

Post-procedurally, the patency of the jailed artery and the stented artery was assessed angiographically. Furthermore, the hemodynamic effect of the implant on aneurysm perfusion was evaluated according to the O'Kelly-Marotta scale [OKM,(19)]. Subsequently, after standardized surveillance on the intensive care unit overnight, cranial computed tomography (CCT) was performed within 24 h after the intervention as a post-interventional standard. Further follow-up examinations were performed in accordance with each center's follow-up regimen.

## Results

### Patients, Aneurysms, and Devices

Overall, 17 patients (mean age: 60.5 years, range: 35–77 years) were included in our study. Slightly more than half of the aneurysms (9/17) were located in the posterior circulation. Two of the patients treated for aneurysms in the posterior circulation had a second aneurysm of the sidewall type, which was treated with the same flow-diverting stent as the aneurysm distant to the parent artery. The dimensions of each target aneurysm, its location, the distance to the remotely implanted flow-diverting stent, the immediate results after implantation of the flow-diverting stent, and the results of follow-up imaging are demonstrated in Table 1. On average, the closest distance between the distal end of the flow-diverting stent and the respective aneurysm neck was 1.65 mm, ranging from 0.4 to 2.4 mm.

### Treatments and Procedural Aspects

**Figure 1:**
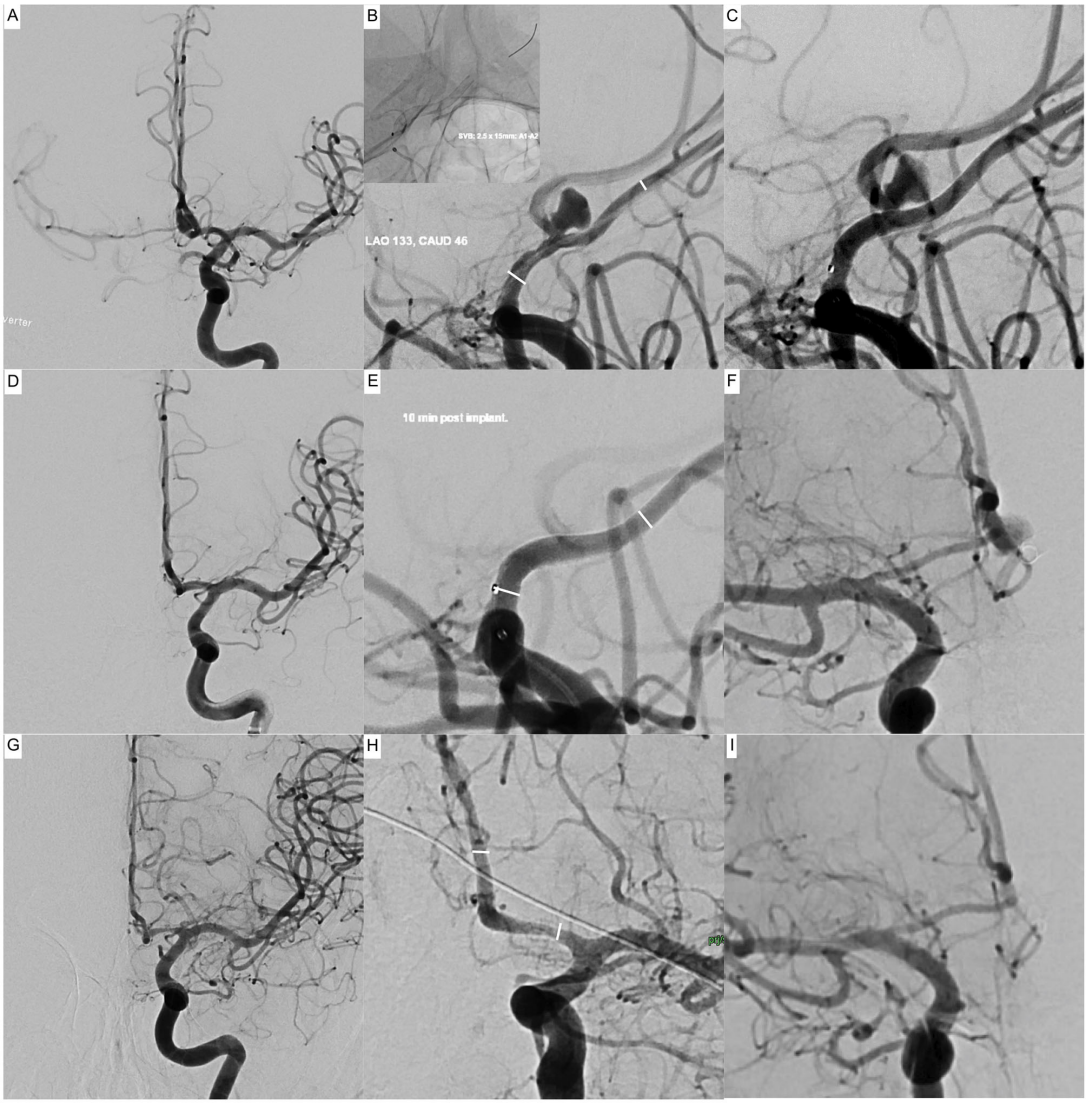
Indirect flow diversion for treatment of an incidental broad-based aneurysm of the anterior communicating artery in a 44-year-old patient. The left A1 segment is dominant; the right A1 segment is hypoplastic (0.7 mm) but contributes significantly to the supply of the ipsilateral anterior cerebral artery territory. The aneurysm (7 × 7 mm fundus, 3.3-mm neck) arises from the middle of the anterior communicating artery (2 mm in diameter). The aneurysm is 2.4 mm distant to the origin of the anterior communicating artery at the A1–A2 junction of the left-hand side. Upper row **(A–C)**: Implantation of a Silk Vista Baby flow-diverting stent into the left A1–A2 segment. **(A)** Initial angiogram of the left-hand side internal carotid artery in posterior-anterior projection prior to implantation. Note the strong crossflow to the contralateral middle cerebral artery via the anterior communicating artery. **(B)** Working projection, prior implantation. The white lines indicate the intended proximal and distal landing zones. The upper left image shows the correspondingly implanted flow diverter. **(C)** Control injection after implantation. The aneurysm dome is already less opacified, indicating a good therapeutic effect. Middle row: **(D–F)** result, 10-min post implantation. **(D)** Despite a forceful injection, there is no more crossflow to the contralateral vessels. **(E)** The anterior communicating artery, including the aneurysm, is no longer opacified. The white lines indicate the proximal and distal endings of the implanted device. **(F)** Injection of the contralateral side: the aneurysm is slightly opacified from the right-hand side A1 segment. Inferior row **(G–I)**: Follow-up angiograms 3 months after treatment. **(G)** Angiogram of the left-hand side internal carotid artery in posterior-anterior projection comparable to **(A)**. The aneurysm is occluded, no crossflow to the contralateral side. **(H)** Magnified image in a slightly oblique projection to visualize the A1–A2 junction. Mild-moderate neointimal hyperplasia at the proximal landing zone. The white lines indicate the proximal and distal endings of the implanted device. **(I)** Angiogram of the right-hand side internal carotid artery in a projection matching. **(F)** The aneurysm is no longer opacified via the contralateral A1.

**Figure 2:**
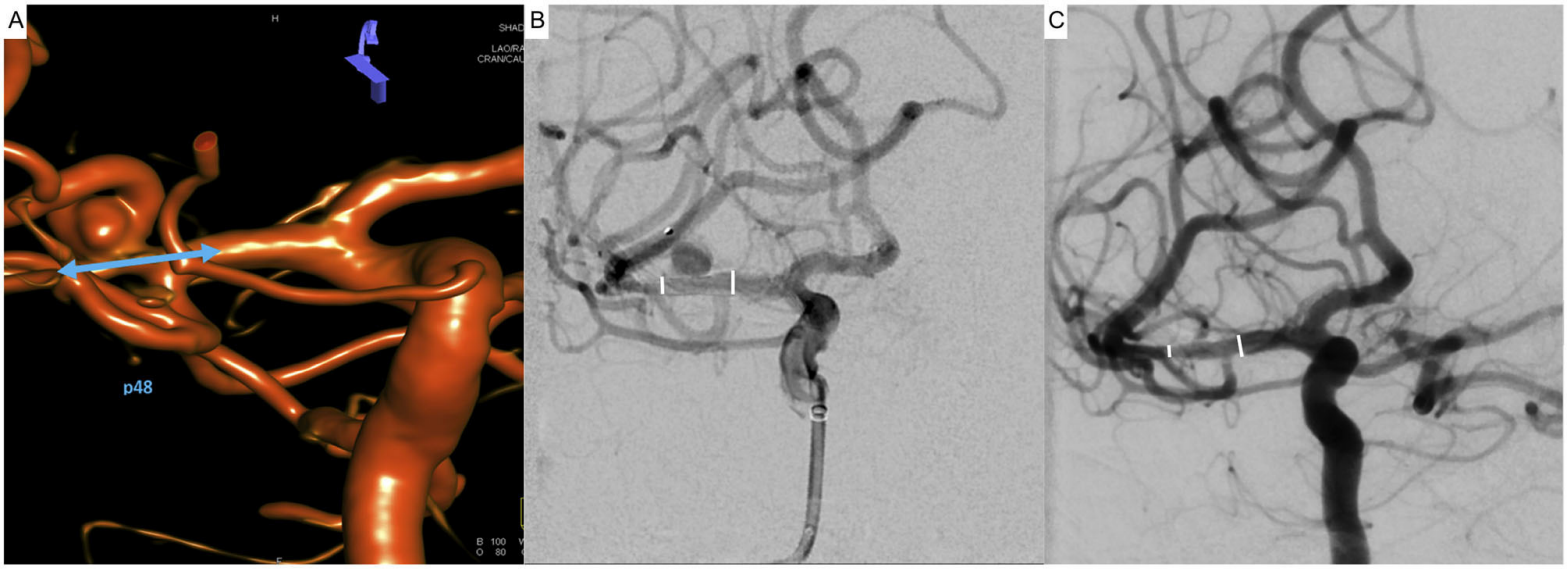
Indirect flow diversion for treatment of an incidental broad-based aneurysm of the right-hand side middle cerebral artery in a 53-year-old patient. The aneurysm (5.6 × 3.4 mm fundus, 4-mm neck) arises from the superior trunk of the middle cerebral artery involving the bifurcation of the latter. The closest distance between aneurysm and outer wall of the treated vessel is 1 mm; however, the aneurysm-parent artery interface is significantly distal to the flow diverter. **(A)** Reconstruction of a 3D rotational angiogram demonstrating the spatial relationship of the aneurysm to the branches of the middle cerebral artery. The aneurysm involves the bifurcation but primarily arises from the superior trunk. The blue arrow indicates the intended proximal and distal landing zones; the goal is to jail the superior trunk and its aneurysm. **(B)** After implantation of the p48MW-HPC flow-diverter stent, jailing the superior trunk and the temporal branch, the control injection revealed prolonged stasis of the contrast agent within the aneurysm (O‘Kelly-Marotta Grade A2). The white lines indicate the proximal and distal endings of the implanted device. **(C)** Four months later, the aneurysm is occluded; all branches of the middle cerebral artery, including the superior trunk, remained patent. The white lines indicate the proximal and distal endings of the implanted device.

**Figure 3:**
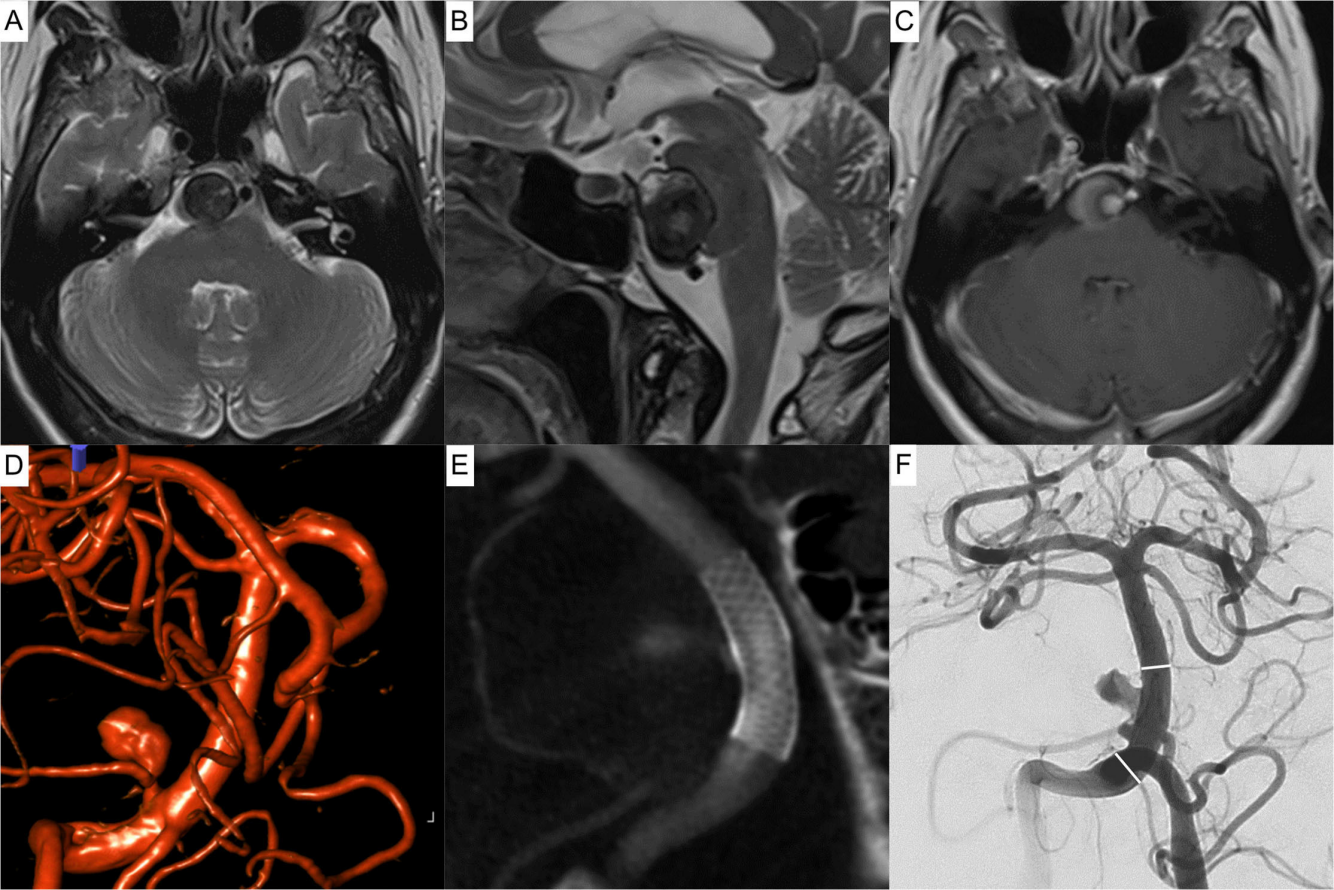
Indirect flow diversion for treatment of a partially thrombosed dissecting aneurysm of the right-hand side anterior inferior cerebellar artery in a 73-year-old patient. The perfused aneurysm (4.5 × 6.3 mm fundus, 2.0-mm neck) is 2.4 mm distant to the basilar artery. Upper row **(A–C)** magnetic resonance imaging of the incidental aneurysm compressing the pons. **(A)** Axial T2 weighted image showing the partially thrombosed aneurysm of the pre-meatal segment of the anterior inferior cerebellar artery lateral to the basilar artery. **(B)** Corresponding sagittal T2 weighted image demonstrating the mass effect of the aneurysm. **(C)** Axial T1 weighted image post Gadolinium showing the basilar artery, the perfused part of the aneurysm and the thrombosed portion. Middle row **(D–F)**: Peri-interventional images. **(D)** Reconstruction of a 3D angiogram prior to treatment, showing the relationship between basilar artery, parent vessel, and aneurysm. **(E)** Contrast enhanced Xper-CT after implantation of a PED Flex Shield 4.75 × 14 mm into the basilar artery, jailing the aneurysm, bearing anterior-inferior cerebellar artery. **(F)** Postinterventional angiogram: aneurysmal perfusion is immediately altered (O'Kelly-Marotta Grade A3). The white lines indicate the proximal and distal endings of the implanted device. At the last available imaging study, 3 months post implantation, the aneurysm discretely decreased in size (not shown).

In total, 17 flow-diverting stents were successfully implanted. Procedural details, together with the applied DAPT regimen, are summarized in Table 1. Figure 1 exemplifies indirect flow diversion in case of a broad based AcomA-aneurysm. Figure 2 demonstrates indirect flow diversion for treatment of an MCA-bifurcation aneurysm. Figure 3 shows indirect flow diversion for treatment of an AICA-aneurysm. None of the cases required more than one flow-diverting stent. Both aneurysms of the right-hand side proximal A1 segment had a claviform shape with a large fundus height and a significant uncoilable neck. To enhance the hemodynamic effect of the flow-diverting stent, the fundus was loosely coiled with a 3D coil. As mentioned above, the patient suffering from the left-hand side PCA aneurysm also had a distinct basilar tip aneurysm, which was treated with a WEB device in the same session. Otherwise, no additional devices or maneuvers including balloon-pta were required to achieve good technical results.

### Unexpected/Adverse Events

#### Unexpected Events Without Clinical Sequelae

In the case of the posterior communicating artery aneurysm treated with a Derivo flow-diverting stent, the latter covered the origins of the posterior- and anterior-communicating arteries of the right-hand side internal carotid artery. After unremarkable implantation of the device, the flow within the covered A1 segment was reduced significantly, as expected. Subsequently, the relationship between the (conflicting) crossflow from the contralateral internal carotid artery *via* the anterior communicating artery and the antegrade flow within the covered right-hand side A1 segment changed toward the disadvantage of the A1 segment, and blood flow stagnated in the latter. Within a waiting period of 15 min, thrombus formed in the proximal A1 segment. A bolus of bodyweight adapted eptifibatide (Integrilin, GlaxoSmithKline) was given intravenously, according to the manufacturer's instructions. The thrombus resolved completely; there were no lesions in diffusion weighted imaging after 24 h. In follow-up imaging after 3 months, the A1 segment remained patent.

In the case of the left-hand side PCA aneurysm involving the atypically originating calcarine artery, an asymptomatic occlusion of the left-hand side ICA occurred at some point during the 4-month follow-up interval. As the left-hand side PcomA was now supplied from the vertebrobasilar territory, flow direction reversed and flow *via* the corresponding PCA increased; the initial hemodynamic effect (OKM Grade A3) was no longer visible in the follow-up after 4 months.

#### Clinical Adverse Events

There were no clinically manifesting complications during or after the treatments.

#### Angiographic Follow-Up

##### Hemodynamic Effect Immediately After Implantation

From a total of 17 assessed aneurysms, the majority (13 lesions) revealed a marked delay in aneurysm perfusion, equivalent to OKM A2-A3 immediately post implantation. In 2 further cases, the aneurysm dome was only partially opacified, corresponding to OKM Grades B1 and B2. Unchanged aneurysm morphology (OKM A1) was observed in 2 cases.

In total, 15/17 aneurysms already showed a distinctly delayed perfusion or even a decrease in aneurysm size immediately after indirect flow diversion.

##### Hemodynamic Effect at the First Follow-Up Imaging

The first angiographic follow-up was available for 12 aneurysms after a mean time of 5.4 months. Of those, one half (6 aneurysms) were already completely excluded from the intracranial circulation, corresponding to OKM D1. One quarter (3 aneurysms) was markedly reduced in size corresponding to OKM B2-B3. Two aneurysms showed significant stagnation within the aneurysm dome (OKM A2-A3). Only one lesion remained morphologically unaltered, equivalent to OKM A1.

In conclusion, after ~5 months, the majority of the treated patients with available follow-ups (11/12 lesions) already showed a distinct delay in aneurysm opacification or even a decreased size of the residually perfused aneurysm dome.

##### Overall Hemodynamic Outcome at the Last Available Follow-Up Imaging

Considering the last available angiographic follow-up of all patients included in this study (*n* = 17), complete aneurysm occlusion (OKM D1) was observed in eight patients. In five patients, the aneurysm dome was only residually perfused, corresponding to OKM B1-B3. Two lesions showed a distinct delay in perfusion with contrast stasis until the venous phase (OKM A3). However, two of the treated lesions remained without appreciable changes in morphology and perfusion despite technically successful flow diversion.

##### Fate of Covered Branches

At the last available follow-up, two of the jailed branches were occluded. The first occluded branch was the AcomA, 3 months after flow-diverter implantation into the dominant A1-A2 segments, as shown in Figure 1. The second occluded branch, also 3 months after the procedure, was the right-hand side A1 segment after implantation of the flow-diverting stent into the ipsilateral M1–communicating ICA. The remaining jailed branches were patent at the last imaging follow-up.

## Discussion

As debated by Dmytriw et al. the uptake of flow-diverting technology in general is rapidly outpacing the availability of clinical evidence, and, especially, evidence on the suitability of flow-diverting technology for treatment of bifurcation aneurysms or anatomically complex lesions is currently insufficient (8). Nevertheless, as a consequence of the broad successful application of flow diversion, humanitarian off-label use has increased tremendously. For example, ruptured dissecting vertebrobasilar aneurysms and aneurysms arising from peripheral cerebral arteries have become recommendable targets for direct flow diversion (9, 10).

Great controversy exists regarding the application of flow-diverting stents for the treatment of bifurcation aneurysms, as the devices not only change the hemodynamic situation in the aneurysm but also alter the perfusion of necessarily covered dependent major branches or perforators (12–16). According to the meta-analysis of Cagnazzo et al. which focused on flow diversion for MCA aneurysms, procedural complications occurred in almost 21% of the cases with persisting deficits in almost 10% and were predominantly related to ischemic events (20). This is contrasted by significantly lower complication rates reported by large-volume neurovascular centers (12–14, 21) and underlines the importance of proper patient selection, treatment technique, and a patient-tailored, appropriate DAPT regimen (18, 22). The results of the latter studies are in line with our experiences with the use of flow-diverting stents for treatment of bifurcation aneurysms, showing good safety and efficacy with low complication rates, especially when applying flow-diverting stents with anti-thrombotic coatings (11, 23).

Although the number of studies on direct flow diversion for treatment of bifurcation aneurysms and side wall-type aneurysms of small caliber cerebral vessels is substantial, comprehensive investigations specifically focusing on the feasibility of indirect flow diversion, i.e., employing flow-diverting stents in a main artery for the treatment of aneurysms arising remotely from small caliber parent arteries, are few. Wajnberg et al. for the first time suggested the approach of progressive deconstruction to treat cerebral aneurysms (24). In this report, a PED was placed across the parent artery of a giant MCA aneurysm, resulting in an asymptomatic occlusion of the aneurysm and its parent vessel over time, compensated by the development of leptomeningeal collaterals. Aguilar-Pérez et al. (18) reported the case of a patient successfully treated with flow-diverting stents for a PICA aneurysm and an aneurysm arising from the second temporal branch of the MCA, where the devices were implanted in the adjacent main arteries (the V4 segment of the vertebral artery and the M1 segment of the MCA) to reduce flow within and subsequently “reconstruct” the small caliber parent arteries. They coined the term “slipstream effect” for the main therapeutic mechanism behind the successful remodeling of the parent artery, despite the aneurysm itself is not covered by the flow-diverting stent. In line with those works, Wallace and coworkers demonstrated the general safety and efficacy of implanting a PED into the vertebral artery, jailing the PICA, for treatment of PICA aneurysms in a series of 14 cases (25). In addition, MacLean et al. demonstrated that implantation of a flow-diverting stent into the PcomA–P2 segment successfully treated two P1 aneurysms by changing the flow within the PcomA–PCA complex, coining the approach “competitive flow diversion” (26). Furthermore, Nossek et al. showed successful treatment of supraclinoid ICA aneurysms and ICA bifurcation aneurysms after disrupting flow in the ipsilateral A1 segments with endovascular techniques, such as flow diversion and coiling (27, 28). In accordance with those reports, the results of our study indicate that indirect flow diversion is a viable approach to aneurysms arising from bifurcations that involve small, efferent branches and aneurysms arising from a peripheral portion of small cerebral arteries as well. The therapeutic effect is based on the progressive deconstruction of the aneurysm and, potentially, its parent vessel (24). Regarding the fate of jailed branches, Iosif et al. demonstrated that the presence of an important collateral supply is decisive for immediate and long-term hemodynamic changes after flow-diverter implantation (29). More specifically, they were able to show that competitive flow from collaterals was associated with an immediate reduction of the flow rate within the covered arteries and, furthermore, led to significantly smaller ostia compared to the control group with absent collateral supply. This finding correlates well with the results in our study—early occlusion of jailed branches only manifested in the presence of competitive flow, for example, after jailing of the AcomA or the A1 segment. As a consequence, careful evaluation of the individual collateral situation at hand is important for treatment success and must be included in the pre-interventional workup. In the light of the aforementioned studies, disconnecting the Circle of Willis with flow-diverting technology, for example, at the ACA-AcomA complex, the PCA-PcomA complex, and the ICA bifurcation, should be considered a functionally significant strategy for aneurysm treatment (26–28).

Nevertheless, a number of uncertainties remain and must be clarified in further studies. First of all, it is important to learn whether there is an efficacy threshold regarding the distance between the flow diverter and the aneurysmal orifice, and other factors like the inflow angle as well as the ratio of diameters of the main artery vs. the parent vessel (30). Furthermore, the most appropriate DAPT combination and its optimal duration must be determined in context of indirect flow diversion. As reflected by recent reports, the choice of the anti-platelet medication is a crucial determinant of the rate of ischemic and hemorrhagic complications in flow diversion (26) and may influence the time point of aneurysm occlusion as well (11). From a current perspective, the combination of ASA and Ticagrelor appears to be superior to ASA and Clopidogrel (22), although single anti-platelet therapy (SAPT) with Prasugrel only has shown promising results in combination with anti-thrombotically covered flow-diverting stents (31). Contrary to that, SAPT using ASA as only anti-aggregant was associated with a significant number of ischemic complications despite the use of flow-diverting stents with anti-thrombotic coating (32). Therefore, a calculated and controlled anti-platelet medication (33), together with a proper selection of the flow-diverting stent with special regards to its hemocompatibility (34) and its flow-diverting potential, certainly influences the success of indirect flow diversion. However, the limited data of our retrospective study are not sufficient to make recommendations in this regard. In general, our study suffers from a number of limitations. The number of included patients is small, and the individual hemodynamic situation of the included cases differs substantially. Procedural and follow-up kinds of imaging were not performed with a specific focus on the aspect of indirect flow diversion; therefore, the relationship between the aneurysm-parent artery interface and the implanted flow diverter is not ideally displayed in a number of cases, underlining the need for an optimized imaging protocol for future studies in this regard. Furthermore, platelet-function testing was not available for most patients, and the occurrence of high on treatment platelet reactivity was, therefore, not evaluable. Also, long-term outcomes and follow-up imaging were not available for a number of included patients; therefore, our results remain preliminary, and validation in a greater patient cohort with long-term follow-ups is required.

## Conclusion

Our study indicates that indirect flow diversion is a safe and feasible approach to the treatment of aneurysms associated with efferent branches of bifurcations and aneurysms arising distantly to the origin of small cerebral vessels. Further studies with long-term follow-ups are needed to validate the concept and to determine the limitations of the approach, especially with regard to the distance between aneurysm and the flow-diverting stent and other hemodynamically important factors like the inflow angle and the ratio of the diameters of the main artery and the parent vessel.

## Data Availability Statement

The original contributions presented in the study are included in the article/supplementary material, further inquiries can be directed to the corresponding author/s.

## Ethics Statement

The studies involving human participants were reviewed and approved by Ethics Committee of the University Hospital Halle (Saale), Germany (IRB00011721 Faculty of Medicine, Martin-Luther-University Halle-Wittenberg). The patients/participants provided their written informed consent to participate in this study.

## Author Contributions

SS performed interventions, was responsible for data acquisition, designed the study, and wrote the paper. RB was responsible for data acquisition, wrote the paper, and performed image analysis. ES performed interventions, data acquisition, and follow-up imaging analysis. MSk and M-SS curated the data and were responsible for vascular analysis. WW and NG reviewed the paper and curated the data. DM, AG, and GB performed interventions, data acquisition, and image analysis. MSc and JP were responsible for data curation and reviewed the paper. VG and MSp performed interventions, image analysis, and reviewed the paper. All authors contributed to the article and approved the submitted.

## Conflict of Interest

SS has proctoring and consultancy agreements with phenox and Balt international. The remaining authors declare that the research was conducted in the absence of any commercial or financial relationships that could be construed as a potential conflict of interest.

## Publisher's Note

All claims expressed in this article are solely those of the authors and do not necessarily represent those of their affiliated organizations, or those of the publisher, the editors and the reviewers. Any product that may be evaluated in this article, or claim that may be made by its manufacturer, is not guaranteed or endorsed by the publisher.
